# Transfusion-Acquired HIV: History, Evolution of Screening Tests, and Current Challenges of Unreported Antiretroviral Drug Use in Brazil

**DOI:** 10.3390/v14102214

**Published:** 2022-10-08

**Authors:** Anna S. Nishiya, Suzete C. Ferreira, Nanci A. Salles, Vanderson Rocha, Alfredo Mendrone-Júnior

**Affiliations:** 1 Fundação Pró-Sangue Hemocentro de São Paulo, São Paulo 05403-000, Brazil; 2 Laboratory of Medical Investigation in Pathogenesis and Targeted Therapy in Oncoimmunohematology (LIM-31), Department of Hematology, Hospital das Clínicas HCFMUSP, Faculdade de Medicina, Universidade de São Paulo, São Paulo 05403-000, Brazil; 3 Disciplina de Ciências Médicas, Faculdade de Medicina, Universidade de São Paulo (FMUSP), São Paulo 05403-000, Brazil; 4 Churchill Hospital, Oxford University, Oxford OX3 7LE, UK

**Keywords:** HIV infection, blood donors, transfusion risk, transfusion-transmission, HIV prophylaxis, window period, antiretroviral, HIV therapy, transfusion safety

## Abstract

Prevention of HIV acquisition by blood transfusion from its emergence to the present day is reviewed, and current challenges are delineated. The experience of Fundação Pró-Sangue/Hemocentro de São Paulo, Brazil, is highlighted in the quest for improvements in blood safety and the evolution of increasingly sensitive and specific screening tests. Concerns and establishing stringent criteria in the screening of potential blood donors are emphasized, and the current criteria for identifying and deferring candidates at high risk of acquiring sexually transmitted diseases are summarized. Future challenges relate to the identification of donors with unreported use of antiretroviral drugs for prophylaxis against possible HIV exposure or for treatment of an HIV infection whose viral expression is undetectable by current analyses. There is a need to better understand the motivation of HIV-exposed donors and to educate them about the risk of transfusion-mediated HIV transmission despite having low or undetectable viral loads. In situations in which traditional HIV RNA or antibody detection assays remain negative, more sensitive analyses are needed to identify potential donors at risk for HIV transmission.

## 1. Worldwide and Brazilian HIV Prevalence

Despite advances in human immunodeficiency virus (HIV)-related prevention, treatment, care, and support, acquired immune deficiency syndrome (AIDS) remains a global crisis. It is estimated that approximately 38.4 million people are currently infected with HIV, of which 1.5 million are recent cases; 650,000 AIDS-related deaths occurred worldwide in 2021 [1]. In Brazil, from 1980 to mid-2021, 1,045,355 cases of AIDS were identified with a detection rate of 14.1/100,000 inhabitants in 2020 [2]. In the last decades, advances in diagnosis and treatment have led to decreases in morbidity and mortality from HIV infection [3]. Approximately 700,000 people living with HIV/AIDS have been treated with antiretroviral drugs (ARVs) that are available without charge in the Brazilian Public Health System. Major treatment regimens include three drugs, tenofovir, lamivudine, and dolutegravir or efavirenz, among other options [4]. Since 2018, the Brazilian Ministry of Health also provides ARVs for pre- or post-exposure prophylaxis to individuals at risk of acquiring HIV infection. More than 39,000 people are currently taking a combination of tenofovir and emtricitabine for HIV prophylaxis [5,6].

## 2. Beginning of the HIV Epidemic and Transfusion-Transmitted HIV

Beginning in the early 1980s, a new disease threatened both recipients of blood products and those responsible for ensuring a safe blood supply [7]. In the period of October 1980–May 1981, the Centers for Disease Control and Prevention (CDC) reported the first five cases of opportunistic *Pneumocystis carinii* pneumonia among young men who had sex with men (MSM) [8]. The cases of *Pneumocystis carinii* pneumonia associated with Kaposi’s sarcoma rapidly increased among homosexual males, and also encompassed heterosexuals who abused IV drugs, as well as Haitian refugees in the United States [9,10]. The underlying cause of this opportunistic infection was given the name AIDS. Evidence of transmission if this disease by blood transfusion was then reported in three cases of *Pneumocystis carinii* pneumonia among individuals with hemophilia “A” who received factor VIII concentrate [11] and who had no other epidemiological risk for the disease. Ammann et al. [12], in 1983, reported a case of an infant who received multiple transfusions during the first few days of life for rhesus disease. The baby developed recurrent infections when 6 months old and subsequently it was found that one of the platelet donors had AIDS. Between 1981 and 1983, more than 2000 patients with this syndrome, and more than 800 deaths from AIDS, were reported [13]. The prevalence of the HIV antibody reached 74% among hemophiliac A patients [14]. Only after discovery of the virus responsible for AIDS [15,16] and development and implantation of HIV antibody screening in 1985, associated with the development of high-risk donor qualification and deferral measures [17,18], was there a reduction in the risk of HIV transmission by blood transfusion. With the recognition of transfusion-associated AIDS as a worldwide threat to blood safety, there was a paradigm shift towards faster implementation of blood safety interventions for all known blood-borne infections and an improved surveillance for emerging pathogens [7,19].

## 3. Donor HIV Screening Tests and Blood Safety Policies in Brazil

In January 1988, a Brazilian federal law established mandatory blood donor registration, as well as the performance of laboratory screening tests for hepatitis B, syphilis, Chagas disease, malaria, and HIV on donated blood, to prevent the transfusion-mediated spread of these diseases [20]. In 1986, prior to this federal regulation, the government of the state of São Paulo had already made mandatory public network testing in hospitals, blood banks, maternity hospitals, and hemotherapy centers of the state to detect antibodies to HIV in the material collected for blood transfusions and/or their derivatives [21]. At Fundação Pro Sangue Hemocentro de São Paulo (FPS_HSP), one of the largest blood banks in Latin America, screening for HIV in blood donors was initiated after the state mandate in 1986.

**Figure 1:**
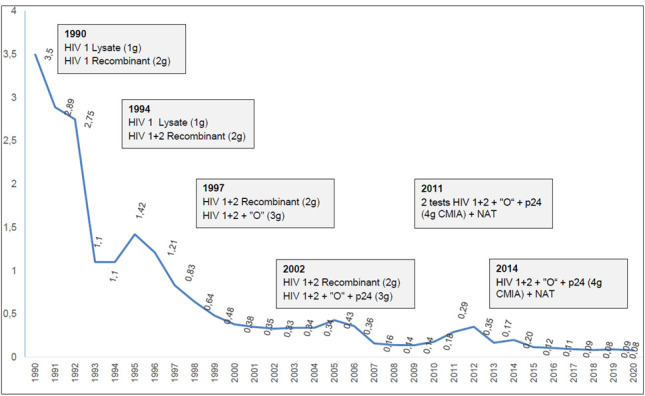
HIV discard rate of different generations of serological kits for blood donor screening tests from 1990 to 2020 and of NAT-HIV from 2011 to 2020 at FPS-HSP (1 g, first generation; 2 g, second generation; 3 g, third generation; 4 g, fourth generation kit; chemiluminescent immunoassay, CMIA).

The complete accumulated data from 1990 to 2020 of the discard rate of donated blood due to HIV serological screening tests are shown in Figure 1. The rate decreased from 3.5% in 1990 to 0.08% in 2020 [22]. In addition to the rejection of donors exposed to or diagnosed with infections, improvements in the clinical interview of donors, and increased reliance on safety-proven repeat donors, the main reason for the decrease in the discard rate was enhanced sensitivity and specificity of HIV detection kits [23,24]. The sensitivity and specificity of first, second, third, and fourth generation tests increased from 99.0%, >99.5%, >99.5%, to >99.9% for sensitivity and from 95.0–98.0%, >99.0%, >99.5%, to 99.5% for specificity, respectively [23]. Since the 1980s, there have been five generations of immunoassays (IAs) using different antigen preparations and detection chemistries for the accurate screening of high-volume samples by blood banks and centralized laboratories. First-generation assays used antigens derived from whole viral lysates present in HIV-positive cultures. In second-generation assays, utilizing advances in molecular biology, synthetic peptides or recombinant proteins derived from immunodominant regions (IDR) of HIV-1 and gp36 proteins of HIV-2 were introduced. Third-generation assays utilized recombinant p24 antigens, HIV-1 group M-derived gp160, a recombinant HIV-2 gp36 IDR peptide, and a synthetic HIV-1 group “O” HIV-1 subtype O peptide [25,26]. Fourth-generation IAs, such as the Abbott Architect HIV Ag/Ab Combo Assay, used fully automated chemiluminescent microparticle technology to simultaneously detect the HIV-1 p24 antigen and antibodies to HIV-1 (groups M, N, and O) and HIV-2. Fifth-generation IAs, such as the Bio-Rad BioPlex 2200 HIV Ag-Ab assay, used multiple sets of magnetic beads coated with p24 monoclonal antibodies and specific epitopes of HIV-1 (groups M, N, and O) and HIV-2 [27].

Currently, a chemiluminescent 4th generation immunoassay for HIV-1/2 antigen/antibody (Abbott Architect) detection is used in the screening of blood donors at FPS-HSP.

## 4. Donor HIV Nucleic Acid Testing (NAT)

Antibody screening alone does not eliminate the risk of transfusion-transmitted HIV infection [28]. NAT further reduced the risk of transfusion-transmitted infection by blood products by shortening the pre-seroconversion detection period [29]. 

In November 2013, screening by NAT for HIV and hepatitis C virus (HCV) became mandatory for all blood donations collected by public and private blood banks in Brazil [30]. At FPS-HSP, NAT for HIV and HCV started in 2011, two years before it became mandatory in the country. Donations during this window period revealed an HIV NAT positivity rate of 3.60 per 1,000,000 donations between 2011 and 2020 [22] that further prevented transfusion-transmitted HIV [31].

Currently, NAT in minipools (MPs) of six samples (Bio-Manguinhos/FIOCRUZ) is used in the screening of blood donors in FPS-HSP.

## 5. Current Intervention Measures to Ensure Transfusion Safety

Blood-transfusion therapy is essential for the management of diverse diseases, and there are approximately 4 million blood donations per year in Brazil [32]. In addition to the testing of all donated blood, the selection and deferral of high-risk donation candidates is a crucial additional strategy employed to reduce the risk of transfusion-transmitted infections. Brazilian law stipulates that all donations must be voluntary and non-remunerated. Prior to blood collection, donors are interviewed to assess potential risk factors. In addition, the donor can opt for confidential unit exclusion (CUE) if they omitted reporting any risk for sexually transmitted diseases [33,34]. The prevalence of HIV detection in first-time donors is higher than in repeat donors [35], so there are concerted efforts to increase repeat donor participation in blood banks. Among initial donors, many expressly seek blood testing over personal concerns of possible infection [35,36]. Current MSM policies were seen as unjustified and discriminatory and led to discussion and pressure by MSM communities for their elimination [37]. Thus, the legislation that deferred donations from MSM and/or were sexual partners of MSM in the preceding 12 months was reversed in Brazil in 2020 and became based on individual practices [33,38]. However, donors who reported engaging in sex with more than three partners, having sex with someone who tested positive for HIV, or sex with an unknown or casual partner are all deferred for 12 months regardless of their sexual orientation [33,38]. Recent recommendations include the deferral of donors who report HIV pre- or post-exposure prophylaxis in the last six months and definitive deferral if receiving ARV therapy to treat HIV [33] practice, already carried out in our blood bank.

## 6. New Challenges in Transfusion-Transmissible HIV Infection

Despite all measures taken to reduce blood-borne infections, there is still a risk of transfusion-mediated transmission of HIV. This mode of transmission was documented to still occur despite the implementation of NAT screening [33,34,35,36,37]. NAT has been implemented in several countries and the sensitivity of the techniques and especially the number of samples in pooled analyses or even in individual NAT can impact the length of the infectious pre-seroconversion window [39,40,41,42,43,44]. The possibility of transfusion-associated HIV transmission is real, even when the viral load is below the limit of detection. The transfusion of HIV-infected blood products despite NAT is described in Table 1. In the United States, the residual risk of transfusion-transmissible HIV infection after NAT is one in two million [19]. In Brazil the estimated residual risk of HIV transmission by transfusion is about 5.46 per 1,000,000 donations [45]. The main risk for transfusion-transmitted HIV infection is utilization of blood obtained from infected donors prior to their HIV seroconversion and NAT detection. A current concern is the failure of blood donors to report their use of ARVs that will inhibit viral replication to undetectable levels, delay seroconversion, and can lead to the loss of previously detectable antibodies (seroreversion) [46,47,48,49]. In Brazil, a country with more than 200,000,000 inhabitants, almost 40,000 individuals use ARVs in prophylaxis and 660,000 in the treatment of their HIV infection [2,4], and its potential to yield false negatives results on HIV screening tests of donated blood remains a serious new risk to transfusion safety [50,51]. The unreported use of ARVs among blood donors in the USA was identified in 15.4% of confirmed HIV-positive patients or 87.3% of those with only a positive serology (HIV-Ab/Ag-positive and HIV-RNA-negative). In these donors, the combination of four drugs (elvitegravir, cobicistat, emtricitabine, and tenofovir, known as Genoya or Stribild) was detected in most cases [50]. In South Africa, utilization of ARVs was detected in 66% of donors who were HIV-Ab-positive/RNA-negative in screening tests, of which 87% were positive for the presence of efavirenz, 8.7% for nevirapine, and 4.7% for lopinavir [51]. In HIV-positive donations, ARV detection was found in 9.8% of the cases, and 94.3% of them tested positive for efavirenz [52]. We have evidence of unreported use of lamivudine among blood donors at FPS-HSP in 2.4% of confirmed HIV-positive patients or 40% of those with only a positive serology (HIV-Ab/Ag-positive and HIV-RNA-negative). We detected low concentrations of lamivudine by high-performance liquid chromatography–tandem mass spectrometry in the plasma of these donors (manuscript in preparation) [53].

ARVs indicated for pre-exposure prophylaxis were identified in 0.6% of first-time male blood donors negative in all screening tests, of which the majority used tenofovir and emtricitabine within 2 days of donating. In addition, 4.8% of HIV-negative MSM donors reported use of prophylaxis close to the time of blood donation [50]. In syphilis-positive donors, 6.5% had detectable levels of tenofovir and/or emtricitabine, but this was not detected in samples from donors who were initially positive for HIV antibodies on screening but who were confirmed as HIV-negative [54].

**Figure 2:**
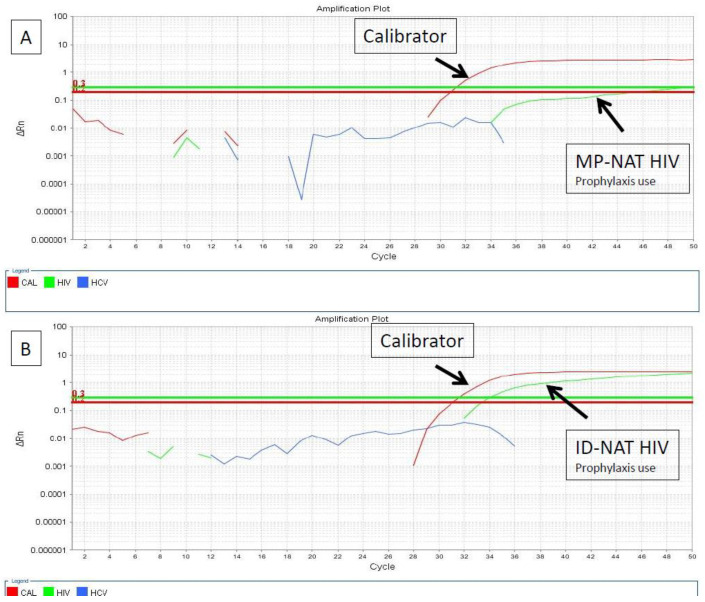
(**A**) The minipooled NAT (MP-NAT-HIV) of 6 samples including from the donor who reported the use of HIV prophylaxis showing an altered curve profile below the cutoff for a positive reaction. (**B**) ID-NAT-HIV from the same donor showing amplification at a high cycle threshold (Ct) value of 34. The HIV reaction curve is shown in green. The calibration reaction curve is shown in red.

We have recently reported a case of blood donation by an individual with unreported HIV prophylaxis and its effect on viral load suppression and a delayed seroconversion [55]. The male donor, 25 years old, provided his second whole blood donation at FPS-HSP in July 2020. His prior donation, negative in all screening tests, was in February 2019. The donor denied any risk for HIV infection or the use of ARVs for treatment or prophylaxis during his clinical screening. He donated 450 mL of whole blood, and his CMIA-HIV antigen/antibody test was negative. However, the minipool of six samples that contained his blood when subjected to HIV NAT showed an altered curve profile despite being below the cutoff for a positive reaction (Figure 2A). A new NAT was performed on individual samples, which showed in this donor amplification at a high cycle threshold (Ct) value of 34 (Figure 2B) and a viral load of less than 10 copies/mL. The laboratory data are presented in Table 2. The donated blood was discarded, and the individual was recalled to the blood bank and invited to provide additional samples. He provided blood three times during the follow-up period. Forty-eight days after the index donation, on August 2020, serological tests (HIV Ag/Ab and Western blot) were negative, and his HIV NAT was also negative. Seroconversion was verified on 10 September 2020, seventy days after the index donation, with positive HIV Ag/Ab and Western blotting tests but with the HIV NAT remaining negative. Following one hundred and five days from his index donation, on October 2020, all HIV-related markers were positive, including the NAT, and the viral load was 151 copies/mL. Recalled donors are routinely re-interviewed, and this donor subsequently admitted using HIV pre-exposure prophylaxis for possible HIV exposure. He reported having sex with a male partner who tested positive for HIV. The individual was referred to the infectious disease department for treatment. Subsequently, his blood sample underwent drug detection testing but was negative, probably due to the irregular use of HIV prophylaxis and/or low sensitivity of the test for tenofovir and emtricitabine [53].

## 7. Strategies to Mitigate These New Challenges

It is evident that people are donating blood who are HIV-positive and on ARVs as well as those who are taking ARV prophylaxis to prevent a possible HIV infection. Even though all potential donors are directly questioned about use of any medication including HIV ARV therapy or HIV prophylaxis, some are not always declaring accurate information in their clinical screening. As shown above, the greatest concern is for those using ARVs who do not yet have levels of HIV antibodies or RNA that are detectable in screening tests. The donor fills out a medical history and interview questionnaire in the initial screening, assessing risk factors for transfusion-transmissible infections, sexual behavior, and also for medication use, among others. Selection through careful interview and clinical evaluation of the donor in addition to donor education reduce the risk of HIV transfusion transmission [56]. Another imminent concern is the use of long-acting injectable drugs for the prevention of HIV infection. Cabotegravir is an integrase inhibitor and can linger in the body for up to 12 months or longer following injection, with a potential effect on donor testing. Thus a two-year deferral plan for injectable cabotegravir is under discussion. More focused and direct efforts to educate blood donation candidates about the risk of unreported ARV use are needed. It should be emphasized that while sexual HIV transmission between partners where one of them is on regular treatment and has an undetectable viral load is unlikely, the same cannot be assumed for blood transfusion. The possibility of transfusion-associated HIV transmission is real, even when the viral load is below the limit of detection. In addition to raising awareness of the window period for HIV detection in donors, initiation of protocols to understand their motivation for blood donations and identification of test-seekers and those seeking primarily to verify the effectiveness of their treatment in controlling the virus will also increase transfusion safety. As repeatedly observed, people using ARVs are not always positive on HIV screening tests, but other markers may indicate elevated risk of transmission. This highlights the importance of surveillance for other sexually transmitted diseases, such as syphilis and hepatitis B virus, in potential blood donors.

Since donors using ARVs may not seroconvert or have a delayed seroconversion and also may have undetectable HIV-RNA by NAT, there is a need for serological and molecular tests of increased sensitivity to improve the screening of donated blood. The use of NAT on individual samples instead of screening pools of blood would be an ideal way to increase sensitivity, but this may not be a viable option due to increased costs. The NAT kit available from the Brazilian government is currently provided for six-sample minipools. Perhaps in the future Brazil will be able to implement individual NAT. Our experience with “in house” (non-commercial) tests typically cost about USD 10–15 per sample. An additional test for samples with undetectable viral loads would be to utilize gene amplification technologies for the detection of HIV proviral DNA that has been integrated into the human genome. This test would provide additional verification of HIV status in inconclusive or suspect samples. Research with drug detection tests is being finalized at our institution, but seems to not currently be feasible due to costs that can reach USD 40–50 per sample and that these tests do not present adequate sensitivity in cases of non-adherence to ARV treatment or prophylaxis. Furthermore, the frequency of ARV use by blood donors can be underestimated due to the short half-life of these drugs in the circulation, which can lead to false-negative drug detection. Further studies are needed to refine and validate these analyses. Finally, a future prospective would encompass the development of protocols to directly treat reticulocytes or whole blood to totally eliminate the presence of HIV or other pathogens.

## Figures and Tables

**Table 1:** HIV serology and molecular test results from HIV-infected blood products despite being negative for transfusion biomarkers.

DATE	Blood Products	HIV Serological Screening	NAT-HIV Screening	NAT-HIV Confirmatory	Pathogen Reduction	Reference
August 2000	Red blood cells	Non-reactive HIV Ab/Ag Abbott	Negative 24 MP-NAT non-commercial LOD 100 cop/mL	180 cop/mL NGI Ultraqual™ LOD 30.76 IU/mL	No	[41]
November 2005	Fresh-frozen plasma and red blood cells	Non-reactive Abbott PRISM Ab-HIV-1/2 assay	Negative 44 MP-NAT—COBAS AmpliScreen LOD 78 IU/mL	135 cop/mL ID-NAT—Procleix Ultrio Assay LOD 20.3 IU/mL	Yes Theraflex	[44]
January 2007	Red blood cells	Non-reactive Abbott PRISM Ab-HIV-1/2 O assay	Negative 96 MP-NAT—COBAS AmpliPrep/TaqMan LOD 4257 IU/mL	146 IU/mL ID-NAT—COBAS AmpliPrep/TaqMan LOD 44 IU/mL	No	[42]
September 2012	Red blood cells	Non-reactive HIV Ag/Ab Combo Architect Abbott	Negative 6 MP-NAT—Biomanguinhos LOD 240 copies/mL	<LOD (7.5 and 3.7 IU/mL) Procleix Ultrio LOD 34.54 IU/mL and COBAS TaqScreen MPX LOD 49 IU/mL	No	[39]
March 2017	Red blood cells	Non-reactive Abbott PRISM Ab-HIV-1/2/O assay	Negative ID-NAT—Procleix Ultrio assay LOD 39 IU/mL (23 copies/mL)	34 copies/mL ID-NAT—COBAS TaqMan LOD 17 copies/mL	Yes INTERCEPT	[40]

NAT, nucleic acid testing; Ab, antibody; Ag, antigen; LOD, limit of detection; MP, minipool; ID, individual.

**Table 2:** HIV serologic and molecular test results over time of donation candidate who reported use of HIV pre-exposure prophylaxis.

DATE	DONATION	CMIA HIV	MP-NAT-HIV	ID-NAT-HIV	RT-PCR HIV	WBlot HIV
		(Cutoff Value of 1.0)			(Copies/mL)	(Positive Bands)
1 July 2020	index	non-reactive	negative	positive	<DL	not conducted
		0.07		ct 34	~0.2	
19 August 2020	return 48 days	undetermined	not performed	negative	negative	undetermined
		0.98				p24
10 September 2020	return 70 days	reactive	not performed	negative	negative	positive
		1.13				p24, gp160
15 October 2020	return 105 days	reactive	not performed	positive	151	positive
		35.4		ct 27.8		p24, gp41, p51
						p66, gp120, and gp160

CMIA, chemiluminescent immunoassay; ct, cycle threshold; DL, detection limit; MP, minipool; ID, individual; WBlot, Western blot; RT-PCR, real-time reverse transcription-polymerase chain reaction.

## References

[1] UNAIDS (2022). In Danger: UNAIDS Global AIDS Update. https://www.unaids.org/en.

[2] Brasil. Ministério da Saúde (2021). Secretaria de Vigilância em Saúde. Boletim Epidemiológico de HIV AIDS. https://www.gov.br/saude/pt-br/centrais-de-conteudo/publicacoes/boletins/epidemiologicos/especiais/2021/boletim-epidemiologico-especial-hiv-aids-2021.pdf.

[3] Maartens G., Celum C., Lewin S.R. (2014). HIV infection: Epidemiology, pathogenesis, treatment, and prevention. Lancet.

[4] Brasil. Ministério da Saúde (2020). Secretaria de Vigilância em Saúde. Departamento de Doenças de Condições Crônicas e Infecções Sexualmente Transmissíveis. Relatório de Monitoramento Clínico do HIV. https://bvsms.saude.gov.br/bvs/publicacoes/relatorio_monitoramento_clinico_hiv_2020.pdf.

[5] Brasil. Ministério da Saúde (2022). Departamento de Condições Crônicas e Infecções Sexualmente Transmissíveis. Painel-Prep. http://www.aids.gov.br/pt-br/painel-prep.

[6] Brasil. Ministério da Saúde (2018). Secretaria de Vigilância em Saúde. Departamento de Vigilância, Prevenção e Controle das infecções Sexualmente Transmissíveis, do HIV/Aids e das Hepatites Virais. Protocolo Clínico e diretrizes Terapêuticas para Profilaxia Pré-Exposição (PrEP) de risco à infecção pelo HIV. https://bvsms.saude.gov.br/bvs/publicacoes/protocolo_clinico_diretrizes_terapeuticas_profilaxia_pre_exposicao_risco_infeccao_hiv.pdf.

[7] Alter H.J., Klein H.G. (2008). The hazards of blood transfusion in historical perspective. Blood.

[8] (CDC) CfDC (1981). Pneumocystis pneumonia—Los Angeles. MMWR Morb. Mortal. Wkly. Rep..

[9] (CDC) CfDC (1982). Update on Kaposi’s sarcoma and opportunistic infections in previously healthy persons—United States. MMWR Morb. Mortal. Wkly. Rep..

[10] (CDC) CfDC (1982). Opportunistic infections and Kaposi’s sarcoma among Haitians in the United States. MMWR Morb. Mortal. Wkly. Rep..

[11] (CDC) CfDC (1982). Pneumocystis carinii pneumonia among persons with hemophilia A. MMWR Morb. Mortal. Wkly. Rep..

[12] Ammann A.J., Cowan M.J., Wara D.W., Weintrub P., Dritz S., Goldman H., Perkins H.A. (1983). Acquired immunodeficiency in an infant: Possible transmission by means of blood products. Lancet.

[13] Reichert C.M., O’Leary T.J., Levens D.L., Simrell C.R., Macher A.M. (1983). Autopsy pathology in the acquired immune deficiency syndrome. Am. J. Pathol..

[14] Gjerset G.F., McGrady G., Counts R.B., Martin P.J., Jason J., Kennedy S., Evatt B., Hansen J.A. (1985). Lymphadenopathy-associated virus antibodies and T cells in hemophiliacs treated with cryoprecipitate or concentrate. Blood.

[15] Barré-Sinoussi F., Chermann J.C., Rey F., Nugeyre M.T., Chamaret S., Gruest J., Dauguet C., Axler-Blin C., Vezinet-Brun F., Montagnier L. (1983). Isolation of a T-lymphotropic retrovirus from a patient at risk for acquired immune deficiency syndrome (AIDS). Science.

[16] Gallo R.C., Salahuddin S.Z., Popovic M., Shearer G.M., Kaplan M., Haynes B.F., Palker T.J., Markham P.D. (1984). Frequent detection and isolation of cytopathic retroviruses (HTLV-III) from patients with AIDS and at risk for AIDS. Science.

[17] Pear R. (1985). AIDS Blood Test to Be Available in 2 to 6 Weeks. http://www.nytimes.com/1985/03/03/us/aids-blood-test-to-be-available-in-2-to-6-weeks.html.

[18] Busch M.P., Young M.J., Samson S.M., Mosley J.W., Ward J.W., Perkins H.A. (1991). Risk of human immunodeficiency virus (HIV) transmission by blood transfusions before the implementation of HIV-1 antibody screening. The Transfusion Safety Study Group. Transfusion.

[19] Busch M.P., Bloch E.M., Kleinman S. (2019). Prevention of transfusion-transmitted infections. Blood.

[20] Brasil (1988). Lei No 7.649, de 25 de Janeiro de 1988. Estabelece a obrigatoriedade do cadastramento dos doadores de sangue bem como a realização de exames laboratoriais no sangue coletado, visando a prevenir a propagação de doenças, e dá outras providências. http://www.planalto.gov.br/ccivil_03/Leis/1980-1988/L7649.htm.

[21] Paulo S., São Paulo Lei N. 5.190, de 20 de Junho de 1986. http://dobuscadireta.imprensaoficial.com.br/default.aspx?DataPublicacao=19860621&Caderno=Poder%20Executivo&NumeroPagina=1.

[22] Salles N.A., Nishiya A.S., de Almeida-Neto C., Ferreira S.C., Rocha V., Mendrone A. (2022). Discard Rate of HIV Serological and Molecular Screening Tests from 1990 to 2020 and HIV NAT Yield Rate between 2011 and 2020.

[23] Alexander T.S. (2016). Human Immunodeficiency Virus Diagnostic Testing: 30 Years of Evolution. Clin. Vaccine Immunol..

[24] Salles N.A., Sabino E.C., Barreto C.C., Barreto A.M., Otani M.M., Chamone D.F. (2003). The discarding of blood units and the prevalence of infectious diseases in donors at the Pro-Blood Foundation/Blood Center of São Paulo, São Paulo, Brazil. Rev. Panam. Salud. Publica.

[25] Zeh C., Oyaro B., Vandenhoudt H., Amornkul P., Kasembeli A., Bondo P., Mwaengo D., Thomas T.K., Hart C., Laserson K.F. (2011). Performance of six commercial enzyme immunoassays and two alternative HIV-testing algorithms for the diagnosis of HIV-1 infection in Kisumu, Western Kenya. J. Virol. Methods.

[26] Louie B., Pandori M.W., Wong E., Klausner J.D., Liska S. (2006). Use of an acute seroconversion panel to evaluate a third-generation enzyme-linked immunoassay for detection of human immunodeficiency virus-specific antibodies relative to multiple other assays. J. Clin. Microbiol..

[27] Salmona M., Delarue S., Delaugerre C., Simon F., Maylin S. (2014). Clinical evaluation of BioPlex 2200 HIV Ag-Ab, an automated screening method providing discrete detection of HIV-1 p24 antigen, HIV-1 antibody, and HIV-2 antibody. J. Clin. Microbiol..

[28] Ward J.W., Holmberg S.D., Allen J.R., Cohn D.L., Critchley S.E., Kleinman S.H., Lenes B.A., Ravenholt O., Davis J.R., Quinn M.G. (1988). Transmission of human immunodeficiency virus (HIV) by blood transfusions screened as negative for HIV antibody. N. Engl. J. Med..

[29] Buschi M.P., Kleinman S.H., Nemo G.J. (2003). Current and emerging infectious risks of blood transfusions. JAMA.

[30] Brasil. Ministério da Saúde (2013). Portaria Nº 2.712, de 12 de Novembro de 2013. Redefine o regulamento técnico de procedimentos hemoterápicos. https://bvsms.saude.gov.br/bvs/saudelegis/gm/2013/prt2712_12_11_2013.html.

[31] Salles N.A., Nishiya A.S., Ferreira S.C., Rocha V.G., Mendrone A. (2019). Detection of HIV-1 infections in blood donors during the pre-seroconversion window period in São Paulo, Brazil. Rev. Soc. Bras. Med. Trop..

[32] Brasil. Ministério da Saúde (2020). Agência Nacional de Vigilância Sanitária—Anvisa. 9o Boletim de produção Hemoterápica, Hemoprod, 2020. Brasília. https://app.powerbi.com/view?r=eyJrIjoiMWM4MDQzNDMtYjZjZC00ZTBhLWFkOTctODdiZjE2ODQ4YTJkIiwidCI6ImI2N2FmMjNmLWMzZjMtNGQzNS04MGM3LWI3MDg1ZjVlZGQ4MSJ9.

[33] Brasil. Ministério da Saúde (2020). Agência Nacional de Vigilância Sanitária—Anvisa. Guia para inclusão de critérios na triagem clínica e epidemiológica de candidatos a doação de sangue baseados em práticas individuais acrescidas de risco para infecções transmissíveis pelo sangue. Guia n° 34/2020—versão 1. https://www.cevs.rs.gov.br/upload/arquivos/202010/19093256-guia-n-34-versao-1.pdf.

[34] Brasil. Ministério da Saúde (2017). Portaria de Consolidação nº 5, de 28 de Setembro de 2017, anexo IV, 2017. Consolidação das normas sobre as ações e os serviços de saúde do Sistema Único de Saúde. http://portalsinan.saude.gov.br/images/documentos/Legislacoes/Portaria_Consolidacao_5_28_SETEMBRO_2017.pdf.

[35] Barreto C.C., Sabino E.C., Gonçalez T.T., Laycock M.E., Pappalardo B.L., Salles N.A., Wright D.J., Chamone D.F., Busch M.P. (2005). Prevalence, incidence, and residual risk of human immunodeficiency virus among community and replacement first-time blood donors in São Paulo, Brazil. Transfusion.

[36] Goncalez T.T., Sabino E.C., Murphy E.L., Chen S., Chamone D.A., McFarland W. (2006). Human immunodeficiency virus test-seeking motivation in blood donors, São Paulo, Brazil. Vox Sang..

[37] Zucoloto M.L., Gonçalez T.T., McFarland W., Custer B., Galdino G., Martinez E.Z. (2019). Blood donation deferral policies among men who have sex with men in Brazil. Hematol. Transfus. Cell Ther..

[38] Brasil. Ministério da Saúde (2020). Agência Nacional de Vigilância Sanitária-Anvisa. Resolução de Diretoria Colegiada—RDC nº399, de 7 de julho de 2020. Revoga a alínea "d" do inciso XXX do art. 25 da Resolução de Diretoria Colegiada—RDC nº 34, de 11 de junho de 2014, que dispõe sobre as Boas Práticas no Ciclo do Sangue, em cumprimento à ordem judicial. https://bvsms.saude.gov.br/bvs/saudelegis/anvisa/2020/rdc0399_7_07_2020.pdf.

[39] Salles N.A., Levi J.E., Barreto C.C., Sampaio L.P., Romano C.M., Sabino E.C., Mendrone A. (2013). Human immunodeficiency virus transfusion transmission despite nucleic acid testing. Transfusion.

[40] Cappy P., Barlet V., Lucas Q., Tinard X., Pillonel J., Gross S., Tiberghien P., Laperche S. (2019). Transfusion of HIV-infected blood products despite highly sensitive nucleic acid testing. Transfusion.

[41] Delwart E.L., Kalmin N.D., Jones T.S., Ladd D.J., Foley B., Tobler L.H., Tsui R.C.P., Busch M.P. (2004). First report of human immunodeficiency virus transmission via an RNA-screened blood donation. Vox Sang..

[42] Schmidt M., Korn K., Nübling C.M., Chudy M., Kress J., Horst H.A., Geusendam G., Hennig H., Sireis W., Rabenau H.F. (2009). First transmission of human immunodeficiency virus Type 1 by a cellular blood product after mandatory nucleic acid screening in Germany. Transfusion.

[43] Vermeulen M., Lelie N., Coleman C., Sykes W., Jacobs G., Swanevelder R., Busch M., van Zyl G., Grebe E., Welte A. (2019). Assessment of HIV transfusion transmission risk in South Africa: A 10-year analysis following implementation of individual donation nucleic acid amplification technology testing and donor demographics eligibility changes. Transfusion.

[44] Álvarez M., Luis-Hidalgo M., Bracho M.A., Blanquer A., Larrea L., Villalba J., Puig N., Planelles D., Montoro J., Roig R. (2016). Transmission of human immunodeficiency virus Type-1 by fresh-frozen plasma treated with methylene blue and light. Transfusion.

[45] Oliveira Garcia Mateos S., Preiss L., Gonçalez T.T., Di Lorenzo Oliveira C., Grebe E., Di Germanio C., Stone M., Filho L.A., Belisario A.R., Custer B. (2021). 10-year analysis of human immunodeficiency virus incidence in first-time and repeat donors in Brazil. Vox Sang..

[46] de Souza M.S., Pinyakorn S., Akapirat S., Pattanachaiwit S., Fletcher J.L., Chomchey N., Kroon E.D., Ubolyam S., Michael N.L., Robb M.L. (2016). Initiation of Antiretroviral Therapy During Acute HIV-1 Infection Leads to a High Rate of Nonreactive HIV Serology. Clin. Infect. Dis..

[47] Hare C.B., Pappalardo B.L., Busch M.P., Karlsson A.C., Phelps B.H., Alexander S.S., Bentsen C., Ramstead C.A., Nixon D.F., Levy J.A. (2006). Seroreversion in subjects receiving antiretroviral therapy during acute/early HIV infection. Clin. Infect. Dis..

[48] Donnell D., Ramos E., Celum C., Baeten J., Dragavon J., Tappero J., Lingappa J.R., Ronald A., Fife K., Coombs R.W. (2017). The effect of oral preexposure prophylaxis on the progression of HIV-1 seroconversion. AIDS.

[49] Kassutto S., Johnston M.N., Rosenberg E.S. (2005). Incomplete HIV type 1 antibody evolution and seroreversion in acutely infected individuals treated with early antiretroviral therapy. Clin. Infect. Dis..

[50] Custer B., Quiner C., Haaland R., Martin A., Stone M., Reik R., Steele W.R., Kessler D., Williamson P.C., Anderston S.A. (2020). HIV antiretroviral therapy and prevention use in US blood donors: A new blood safety concern. Blood.

[51] Sykes W., Van den Berg K., Jacobs G., Jauregui A., Roubinian N., Wiesner L., Maartens G., Custer B., Busch M., Jentsch U. (2019). Discovery of False Elite Controllers: HIV Antibody-Positive RNA-Negative Blood Donors Found To Be on Antiretroviral Therapy. J. Infect. Dis..

[52] van den Berg K., Vermeulen M., Louw V.J., Murphy E.L., Maartens G. (2021). Undisclosed HIV status and antiretroviral therapy use among South African blood donors. Transfusion.

[53] Nishiya A.S., Salles N.A., de Almeida-Neto C., Ferreira S.C., Nogueira F.A.H., Rocha V., Mendrone-Junior A. (2022). Confirmed Use of Antiretroviral Drugs for HIV Therapy in Two Blood Donors with Potential Elite Controller Profile from São Paulo.

[54] Harvala H., Reynolds C., Ijaz S., Maddox V., Penchala S.D., Amara A., Else L., Brailsford S., Khoo S. (2021). Evidence of HIV pre-exposure or post-exposure prophylaxis (PrEP/PEP) among blood donors: A pilot study, England June 2018 to July 2019. Sex Transm. Infect..

[55] Nishiya A.S., Salles N.A., de Almeida-Neto C., Witkin S.S., Ferreira S.C., Nogueira F.A.H., Facincani T., Rocha V., Mendrone A. (2021). Influence of unreported HIV prophylaxis on the kinetics of post-blood donation HIV seroconversion. Transfusion.

[56] Busch M.P. (2022). Four decades of HIV and transfusion safety: Much accomplished but ongoing challenges. Transfusion.

